# Poor Clinical Outcomes for HIV Infected Children on Antiretroviral Therapy in Rural Mozambique: Need for Program Quality Improvement and Community Engagement

**DOI:** 10.1371/journal.pone.0110116

**Published:** 2014-10-20

**Authors:** Sten H. Vermund, Meridith Blevins, Troy D. Moon, Eurico José, Linda Moiane, José A. Tique, Mohsin Sidat, Philip J. Ciampa, Bryan E. Shepherd, Lara M. E. Vaz

**Affiliations:** 1 Vanderbilt Institute for Global Health, Vanderbilt University School of Medicine, Nashville, Tennessee, United States of America; 2 Department of Pediatrics, Vanderbilt University School of Medicine, Nashville, Tennessee, United States of America; 3 Department of Biostatistics, Vanderbilt University School of Medicine, Nashville, Tennessee, United States of America; 4 Department of Preventive Medicine, Vanderbilt University School of Medicine, Nashville, Tennessee, United States of America; 5 Department of Medicine, Vanderbilt University School of Medicine, Nashville, Tennessee, United States of America; 6 Friends in Global Health, Quelimane and Maputo, Mozambique; 7 School of Medicine, Universidade Eduardo Mondlane, Maputo, Mozambique; Johns Hopkins Bloomberg School of Public Health, United States of America

## Abstract

**Introduction:**

Residents of Zambézia Province, Mozambique live from rural subsistence farming and fishing. The 2009 provincial HIV prevalence for adults 15–49 years was 12.6%, higher among women (15.3%) than men (8.9%). We reviewed clinical data to assess outcomes for HIV-infected children on combination antiretroviral therapy (cART) in a highly resource-limited setting.

**Methods:**

We studied rates of 2-year mortality and loss to follow-up (LTFU) for children <15 years of age initiating cART between June 2006–July 2011 in 10 rural districts. National guidelines define LTFU as >60 days following last-scheduled medication pickup. Kaplan-Meier estimates to compute mortality assumed non-informative censoring. Cumulative LTFU incidence calculations treated death as a competing risk.

**Results:**

Of 753 children, 29.0% (95% CI: 24.5, 33.2) were confirmed dead by 2 years and 39.0% (95% CI: 34.8, 42.9) were LTFU with unknown clinical outcomes. The cohort mortality rate was 8.4% (95% CI: 6.3, 10.4) after 90 days on cART and 19.2% (95% CI: 16.0, 22.3) after 365 days. Higher hemoglobin at cART initiation was associated with being alive and on cART at 2 years (alive: 9.3 g/dL vs. dead or LTFU: 8.3–8.4 g/dL, p<0.01). Cotrimoxazole use within 90 days of ART initiation was associated with improved 2-year outcomes Treatment was initiated late (WHO stage III/IV) among 48% of the children with WHO stage recorded in their records. Marked heterogeneity in outcomes by district was noted (p<0.001).

**Conclusions:**

We found poor clinical and programmatic outcomes among children taking cART in rural Mozambique. Expanded testing, early infant diagnosis, counseling/support services, case finding, and outreach are insufficiently implemented. Our quality improvement efforts seek to better link pregnancy and HIV services, expand coverage and timeliness of infant diagnosis and treatment, and increase follow-up and adherence.

## Introduction

Mozambique is one of the most HIV-affected countries with an estimated national HIV prevalence in 2009 of 11.5%, translating into approximately 1.4 million adults living with HIV. [1], [2] Because of its heavy HIV burden, Mozambique is a priority nation for support from the U.S. President’s Emergency Plan for AIDS Relief (PEPFAR). [3] National combination antiretroviral therapy (cART) coverage is low; only an estimated 52% of adults and 20% of children in need of cART were believed to be receiving it as of the end of 2011. [4] In 2010, an estimated 70.8% of pregnant women in their first antenatal care appointment received HIV counseling and testing and 40.2% of HIV-infected pregnant women received ARV prophylaxis for the prevention of mother-to-child transmission (PMTCT), typically single-dose nevirapine [5].

Under 5 (U5) mortality rates have been falling rapidly in Mozambique with 2011 national U5 mortality estimated at 135/1000 live births compared to 219/1000 live births in 1990, in large part because of improvements in vaccination coverage and efforts to manage childhood diarrheal and acute respiratory illnesses. [6] The most recent national estimate is 97/1000 live births, from the latest Demographic Health Survey [7], still shy of the 2015 Millennium Development Goal of 73 deaths per 1000 live births. HIV/AIDS contributes 10% to the U5 mortality nationally [6].

Zambézia Province is a very low-income region of 4.2 million persons in north-central Mozambique whose majority of residents are rural subsistence farmers and fishermen. Zambézia has the nation’s second largest provincial population, representing ≈20% of Mozambique’s total. [1], [8] Provincial HIV prevalence among adults 15–49 years in 2009 was estimated at 12.6% overall, 15.3% among women and 8.9% among men, all higher than national averages, e.g., 13.1% for women 15–49 nationally. [1] Current U5 mortality estimates show Zambézia as having the worst U5 mortality of all provinces, with deaths estimated at 142/1000 live births. [9] Leading causes of U5 deaths in Zambézia in 2009 include neonatal deaths (26.1%), malaria (27.7%) and acute lower respiratory infection (13.7%). HIV/AIDS-related deaths account for 11.5% of U5 deaths in the province [9], [10].

Children with HIV are not in care at as high a proportion as adults with HIV in Africa, and their outcomes are not as good in most programs. [11]–[71] To assess mortality for HIV-infected children on cART, we reviewed data from PEPFAR-supported clinics run by the Zambézia Provincial Health Directorate (Direcção Provincial de Saúde, DPS) in 10 districts where a Vanderbilt University non-governmental organization (NGO) provides technical assistance.

## Methods

Both the Mozambican National Bioethics Committee for Health (*Comité Nacional de Bioética em Saúde* [CNBS]) and the Institutional Review Board of Vanderbilt University approved this analysis. Analysis was performed on routinely collected, de-identified, aggregate patient level data and no individual informed consent was obtained. The CNBS and the Vanderbilt Institutional Review Board explicitly waived the need for written informed consent from the participants.

We analyzed data from a cohort of HIV-infected children <15 years of age initiating cART between June 2006–July 2011 in 10 of 17 rural districts in Zambézia Province. Details of our Friends in Global Health NGO clinical program with the DPS and the Ministry of Health (Ministério de Saúde [MISAU]) have been reported previously. [72] Two districts for which we were responsible did not have electronic medical records at the time of analysis and so were excluded;[73] five additional districts were supported by another NGO in this time period. [74] Patients who transferred from another facility after starting cART were not included in this analysis as data on their care history was incomplete (N = 156).

Patient characteristics at treatment initiation of those alive, lost, and dead at the end of 2 years’ follow-up were compared using rank sum and chi-square tests. Deaths were ascertained from both clinical records and from parental testimonials. Mozambican national guidelines define loss to follow-up (LTFU) as no effective clinical contact within 60 days after the last scheduled medication pickup. [75] Two additional definitions of LTFU from the literature were also applied for the purpose of cross-cohort comparisons. The ‘universal’ definition classifies patients as LTFU if there is no effective clinical contact within 180 days of database closure. [76] The ‘reference’ definition assigns 1 day of follow-up to any individual who does not return following treatment initiation, includes only individuals initiating ART 6 months prior to the database closure, and classifies patients as LTFU if there is no effective clinical contact within 180 days of database closure. [77] All three LTFU definitions deem the patient lost at the date of last contact as opposed to the date of missed visit. Kaplan-Meier estimates were used to compute mortality and the combined endpoint of mortality and LTFU. Cumulative incidence of LTFU was calculated by treating death as a competing risk. Mortality estimates assumed non-informative censoring, i.e., patients LTFU were assumed to have rates of death similar to patients not LTFU. This likely implies that our mortality calculations are under-estimates of true mortality.

Our study did not include children enrolled in HIV care who never initiated treatment. [78] Cotrimoxazole (CTX) data were treated as a tick box for “yes”, collected each visit for the corresponding visit date. If a patient was on CTX anywhere from 0 to 365 days before ART initiation, we considered the patient as “CTX use prior to cART”. If a patient was on CTX anywhere from 90 days before to 90 days after ART initiation, we considered the patient as “current CTX use”.

## Results

During five years of PEPFAR support, 753 HIV-infected children <15 years of age initiated cART. Of these children, 678 (90.0%) were <8 years of age at cART initiation, 397 (52.7%) were <2 years, and 191 (25.4%) were <1 years. Girls represented 57% of the pediatric cART patients. Median CD4+ T-lymphocyte cell count (CD4 counts) and percentage (CD4 percentage) at cART initiation were 497 and 15, respectively, although these quantities were missing for 62% and 70% of patients. Nearly half (48%) of children initiated cART very late in their disease progression (WHO stage III or IV), although WHO stage was missing for 58% of patients.

**Figure 1:**
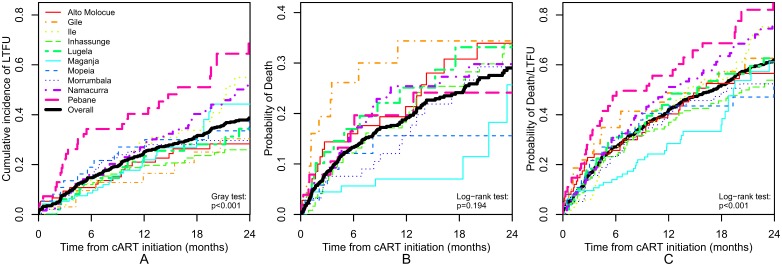
Variation by district in pediatric loss to follow up (LTFU), death, and death or LTFU for 2 years following combination antiretroviral therapy initiation, 10 districts of Zambézia Province, Mozambique, 2006–2011.

Two years after cART initiation, 152 patients had died and 240 were LTFU. At two years, the estimated probability of death was 29.0% (95% confidence interval [CI] 24.5–33.2), the cumulative incidence of LTFU was 38.7 (95% CI 34.8–42.9), and the probability of either death or LTFU was 62.0% (95% CI 57.6–65.9). We observed substantial heterogeneity between districts in two-year outcomes (Figure 1). Two year LTFU ranged from a district low of 25% to a high of 70% (Fig. 1A; p<0.001), mortality ranged from 16–34% (Fig. 1B; p = 0.19), and death or LTFU ranged from 51.1–88.1% (Fig. 1C; p<0.001). The association between treatment duration and mortality rate did not suggest a marked decline in mortality over time. At 90 days on cART, the mortality rate was 8.4% (95% CI: 6.3, 10.4). At 365 days on cART, the mortality rate was 19.2% (95% CI: 16.0, 22.3) and at 730 days (two years) on cART, the mortality rate was 29.0% (95% CI: 24.5%, 33.2%). Cumulative incidence of LTFU was lower when applying two definitions from the literature. [75] The cumulative incidence of LTFU using the ‘universal’ definition was 26.0% (95% CI 22.6–29.9) at 2 years. [76] The cumulative incidence of LTFU using the ‘reference’ definition was 26.4% (95% CI 22.9–30.2) at 2 years [77].

Table 1 compares patient characteristics at cART initiation between those who were alive, dead, and lost after two years. We did not detect any difference in CD4 counts or percentage at cART initiation in children who were alive and on treatment at 2 years compared to those who were either not alive or not in care at that time (p = 0.6), though we had high rates of missing data. We observed higher hemoglobin at the time of cART initiation among those children alive and on treatment at 2 years (alive: median 9.3 g/dL; 8.3 for dead; 8.4 g/dL for LTFU, p<0.01 for alive vs. dead or LTFU). Any cotrimoxazole use in the year prior to ART initiation was associated with improved 2-year outcomes (alive: 76%, dead: 49%, lost: 55%). Cotrimoxazole use within 90 days of ART initiation was associated with improved 2-year outcomes (alive: 69%, dead: 58%, lost: 59%).

**Table 1:** Characteristics of children at initiation of combination antiretroviral therapy by 2 year outcome in 10 districts of Zambézia Province, Mozambique, 2006–2011 (PITC = Provider-initiated testing and counseling; PMTCT = Prevention of mother-to-child HIV transmission; BMI = Body Mass Index or weight in kg divided by height squared).

	Alive	Dead	Lost	Combined	P-value
	(n = 361)	(n = 152)	(n = 240)	(n = 753)	
Female, n(%)	204 (57%)	73 (48%)	118 (49%)	395 (52%)	0.1
Age (years), median (IQR)	2 (1, 4)	1 (0, 2)	1 (1, 3)	1 (0, 4)	<0.001
District, n(%)					0.002
Alto Molócuè	38 (11%)	19 (12%)	19 (8%)	76 (10%)	
Gilé	15 (4%)	10 (7%)	8 (3%)	33 (4%)	
Ile	23 (6%)	10 (7%)	22 (9%)	55 (7%)	
Inhassunge	69 (19%)	31 (20%)	30 (12%)	130 (17%)	
Lugela	22 (6%)	13 (9%)	15 (6%)	50 (7%)	
Maganja	58 (16%)	9 (6%)	28 (12%)	95 (13%)	
Mopeia	19 (5%)	5 (3%)	13 (5%)	37 (5%)	
Morrumbala	52 (14%)	18 (12%)	26 (11%)	96 (13%)	
Namacurra	47 (13%)	28 (18%)	50 (21%)	125 (17%)	
Pebane	18 (5%)	9 (6%)	29 (12%)	56 (7%)	
Referral site^4^, n(%)					0.7
Missing	297 (83%)	134 (88%)	193 (80%)	624 (83%)	
External consultation (PITC)	10 (16%)	4 (22%)	6 (13%)	20 (16%)	
Medical inpatient (PITC)	2 (3%)	0 (0%)	4 (9%)	6 (5%)	
Tuberculosis care (PITC)	1 (2%)	0 (0%)	0 (0%)	1 (<1%)	
PMTCT site	7 (11%)	3 (17%)	8 (17%)	18 (14%)	
Voluntary counseling andtesting site	44 (69%)	11 (61%)	29 (62%)	84 (65%)	
Height (cm), median (IQR)^1^	85 (67, 109)	70 (63, 84.2)	72 (66, 81.8)	75 (66, 103.5)	0.04
Missing	206 (57%)	118 (78%)	174 (72%)	498 (66%)	
Weight (kg), median (IQR)^1^	8.5 (6.3, 14)	6.7 (5, 9.5)	7 (5.8, 10)	7.5 (6, 12.4)	<0.001
Missing	4 (1%)	10 (7%)	24 (10%)	38 (5%)	
BMI (kg/m^2^), median (IQR)^1^	15.2 (14.1, 16.6)	14.4 (13.8, 17.4)	14.9 (14.2, 16.6)	15.1 (14.1, 16.8)	1
Missing	274 (76%)	132 (87%)	197 (82%)	603 (80%)	
CD4+ cell count/µL, median (IQR)^2^	458 (248, 760)	595 (164, 734)	513 (314, 841)	497 (237, 774)	0.7
Missing	222 (61%)	90 (59%)	149 (62%)	461 (61%)	
CD4 percentage, median (IQR)^2^	15 (10, 21)	15 (12, 22)	15 (8, 21)	15 (10, 21)	0.6
Missing	251 (70%)	107 (70%)	171 (71%)	529 (70%)	
Hemoglobin (g/dL), median (IQR)^2^	9.3 (8, 10.4)	8.3 (7, 9.3)	8.4 (7.4, 9.4)	8.9 (7.6, 9.9)	<0.001
Missing	237 (66%)	100 (66%)	163 (68%)	500 (66%)	
WHO stage, n(%)^2^					0.1
Missing	224 (62%)	75 (49%)	130 (54%)	429 (57%)	
I	47 (34%)	18 (23%)	32 (29%)	97 (30%)	
II	30 (22%)	11 (14%)	29 (26%)	70 (22%)	
III	43 (31%)	39 (51%)	37 (34%)	119 (37%)	
IV	17 (12%)	9 (12%)	12 (11%)	38 (12%)	
Cotrimoxazole use (prior to ART), n(%)^3^	273 (76%)	75 (49%)	132 (55%)	480 (64%)	<0.001
Cotrimoxazole use (current), n(%)^3^	249 (69%)	88 (58%)	142 (59%)	479 (64%)	0.01

Percentages are computed using the number of patients with a non-missing value.

1 Weight, height, and BMI are collected at enrollment. ^2^Collected within 90 days before and 14 days after ART initiation. ^3^Prior to ART means any cotrimoxazole (CTX) use recorded in 365 days prior to ART initiation. Current means any CTX use in 90 days before or 90 days after ART initiation. CTX use is recorded along with the visit date; data is not collected on non-users so we are unable to assess missing data. ^4^When PITC referral sites are grouped: p = 0.9.

## Discussion

The experience from our PEPFAR cART program found that 29% of children initiating cART were dead within two years. It is likely that many of the 39% LTFU are at high risk of death or have already died. HIV care for children is not yet optimized in this impoverished setting with a backdrop of health workforce shortages, poor health care infrastructures, challenging transportation, poor maternal and child health outcomes, high rates of tuberculosis and malaria infections, high levels of malnutrition, low adult and pediatric cART and maternal ARV prophylaxis coverage rates, and limited formal counseling/social support programs. Similar challenges are reported elsewhere, particularly where cART is initiated late and co-infections are already extant. [20], [79], [80] Considerably better outcomes are reported from LMIC outside of Africa. [81]–[92] While a number of pediatric cART programs have reported much better success, we do not know the extent to which there is a reporting bias in the literature, i.e., overrepresentation in the literature of more favorable program outcomes. [11], [44], [60], [64], [65], [68], [69], [85], [93]–[110] Challenges we face have been reported from many low and middle-income nations, though we think our results are especially worrisome [25], [111]–[117].

We observed a wide range of LTFU in different districts, suggesting possible inconsistent fidelity across sites to the active case-finding (*busca activa*) program that is in place, as well as variations in the quality of care, system infrastructure and/or community engagement. [118] In PMTCT work in Zambia and subsequently in the multinational PEARL study, similar clinic-by-clinic diversity has been seen, documenting that the specific component of the continuum of care that is “broken” in lower functioning clinics may differ by clinic [119]–[123].

In a poorly functioning clinic in Mozambique, we may find health providers who are able to speak only Portuguese with clients, rather than the local language. We have learned from pediatric and obstetric quality improvement work that mothers frequently do not understand complex instructions in Portuguese from health providers who often come from other provinces and may not speak one of the local languages spoken in this ethnically diverse province. [8], [118], [124] On aggregate, Zambézia residents have low health literacy and numeracy rates, likely contributing to patient/caregiver-provider miscommunication and, possibly to LTFU and suboptimal adherence [125].

Another common occurrence in a poorly functioning clinic generally is the failure of pyschosocial services to effectively engage caregivers fully in chronic pediatric care services, as well as HIV services for themselves. Prior to HIV services, long-term follow-up of chronic diseases was not something with which residents of Zambézia Province were familiar. It is common, especially in rural Africa, that asymptomatic or improving children, parents or guardians do not recognize the need for ongoing services. [126]–[132] The same applies to parents themselves; they may be LTFU once they feel better. [72], [78] We have also had anecdotal reports of parents in our program avoiding care for their children (or themselves) due to stigma and fear of persons learning of the HIV infections in their children. These are daunting challenges that call for more effective counseling and trust-building between providers and clients, and potentially for earlier engagement of children in their health care. The active case-finding approach (*busca active*) of the DPS/MISAU needs serious review and improvement in the face of high mortality and LTFU data in children. It is also possible that traditional active case-finding efforts need to be tailored for special populations, such as children. Improved counseling and family-centered treatment approaches need further exploration. Any innovation in engaging HIV-infected women in their own care can be expected to improve follow-up for their children as well. [133]–[135] A recent review found that although there is evidence of effectiveness of interventions to improve access and adherence to cART, there is less known about major barriers and ways to address them among vulnerable groups such as women, children and adolescents [136].

There is little tradition of long-term pediatric care in rural Zambézia Province. Mothers take children for vaccines and acute illnesses, but only a tuberculosis diagnosis results in chronic care involving long-term drug administration that can reasonably be expected to be available (such medications as insulin and oncology drugs are not available in the rural clinics). In fact, loss to follow-up rates for children with HIV are high throughout southern Africa. [137] Mothers have told us that they and/or their fathers do not want the stigma of having them take the child for HIV care, that they live too far away and cannot afford the time or money for care, they do not know the health workers due to high turnover rates, and that health workers often mistreat them and violate their confidentiality and their privacy [124], [138], [139].

There is evidence from this study and elsewhere that early infant diagnosis, provider initiated testing and counseling, case finding of older children, and family support and outreach are not sufficiently developed in rural Mozambique. [8], [73], [78], [118], [124], [125], [134], [135], [140]–[152] As of 2011, all HIV-infected children <2 years of age should be started on cART as per Mozambican national guidelines, based on results of the South African CHER trial. [79] As of May 2013, Mozambican guidelines changed further to mandate cART for all infected children <5 years of age, independent of clinical status or CD4+ cell count. Yet our study suggests that poor adherence by health workers to standards of screening and HIV staging and subsequent CD4 monitoring impairs pediatric outcomes by delaying recognition of children in need of cART and prophylaxis for opportunistic infections (OI). OI prophylaxis with cotrimoxazole was a protective factor for adverse outcomes in our study. We do not believe that co-trimoxazole benefits are explained by urban-rural differences, as all our sites were rural, nor by family income or assets. All HIV-related services provided by the Ministry, which includes all of the services in this study, are available free of cost, including provision of cotrimoxazole. Family income is not recorded on the clinical record, only patient (or parent) profession; we are thus unable to distinguish subsistence farmers from those who sell their crops, small merchants from larger ones. Over 80 percent of the overall population in the province subsists on less than USD 2 per day as we have documented in a baseline USAID report (Vergara, AE, Blevins M, Vaz LME, et al (2011). Baseline survey report: Improving livelihoods and health of children, women and families in the Province of Zambézia, Republic of Mozambique (available at [http://globalhealth.vanderbilt.edu/programs/scip/]).

Since many children are not diagnosed early or begun on cART early and/or fail to stay in (or adhere to) cART-based care, adverse events are high. [118] We believe that poor interpretation of the guidelines by providers and an overall reluctance to place young children on cART is playing a major role. Quality improvement efforts are essential [148] and are underway to improve infant diagnosis and treatment initiation. [124], [135] Linkages across MCH services are being forged to improve treatment outcomes.

Health worker shortages contribute to poor quality of pediatric care. Given severe health care worker shortages and structural impediments to effective long-term care services, we believe that international support, such as that available from PEPFAR and the Global Fund to Fight AIDS, Tuberculosis and Malaria, will be needed for many years to come. [153]–[157] Whether traditional healers, far more numerous than allopathic practitioners, can be engaged in a productive way for early referral and for assistance in adherence and follow up is unknown. [158], [159] More effective community engagement is essential and some success has been had with church-based outreach. [159], [160] It is also unknown the extent to which efforts such as the Medical Education Partnership Initiative,[161] the Royal Society-DFID Africa Capacity Building Initiative,[162] or the Consortium of New Southern African Medical Schools [163] will make a major difference over the next 5–10 years in addressing chronic health worker shortages in rural Africa. [164]–[167] Task-shifting would be a reasonable approach, but nursing and medical assistants (*técnicos de medicina* in Mozambique) are also in very short supply. [8] Creative approaches to patient-to-patient adherence and retention show promise [168]–[170].

Our data have limitations that affect the completeness of our study. Missing data were frequent, particularly CD4 counts, CD4%, and WHO stage, limiting our ability to examine delays in initiation of treatment. Multivariable analyses were not performed to estimate independent associations with clinical outcomes due to large amounts of missing data and potential for misclassification among those LTFU. We only ask age (in years) of the child such that age subgroups are less reliable, particularly for younger ages, than if we had reliable birthdate; however, rural populations often do not know specific birthdates. Information on cotrimoxazole use was recorded; however, the nature of the documentation is to record use and thus we are unable to differentiate between non-users and missing data. If data are not missing completely at random, there would be bias in the summary statistics of non-missing data. Generalizability of findings to the whole province was limited because data were available for 10 of the total 17 districts. Our database does not collect information on risk factors for poor clinical outcomes external to the patient visit; prospective data collection on such factors (e.g., health facility staffing, drug stock-outs, family support) would permit more robust risk assessment. Nonetheless, we believe that our data clearly indicate a seriously underperforming pediatric care program in need of aggressive quality improvement; despite limitations, we have found these real-world data to be adequate to guide programmatic improvement and community engagement. These efforts are beginning to bear fruit [124], [135], [148].

There are many challenges not likely to be resolved soon: health care worker shortages and high turnover rates, particularly in remote rural settings, drug and supply stockouts, language barriers, gender-power distortions, literacy and numeracy challenges, poor attitudes of health care workers towards patients, lack of appreciation of the germ theory of disease, crushing rural poverty, poor transportation infrastructures, and structural barriers within the clinical care setting. [73], [78], [146], [148], [153], [171]–[173] Co-infections prevalent in the tropics and food shortages are recurring challenges that are far less prevalent in higher income nations. [174]–[177] Drug resistance has not been studied widely in Mozambique [178].

Even in the face of these obstacles, we and others are having some success in pediatric care HIV quality improvement. [124], [135], [179]–[181] That our real-world findings of co-trimoxazole benefit to children in HIV care reinforces clinical trial results suggesting that HIV-infected children benefit from continued co-trimoxazole (protecting against both malaria and non-malarial disease), even when they are on cART. [182] To better retain children on cART and co-trimoxazole, more comprehensive quality improvement efforts are needed to identify staff, structural, cultural, social and policy challenges and to craft solutions for support to pediatric patients, their caregivers, and health care providers.
